# A Competitiveness-Based Theoretical Framework on the Psychology of Income Inequality

**DOI:** 10.1177/09637214231159563

**Published:** 2023-04-27

**Authors:** Nicolas Sommet, Andrew J. Elliot

**Affiliations:** 1 LIVES Centre, University of Lausanne; 2 Department of Psychology, University of Rochester

**Keywords:** income inequality, perceived and personal competitiveness, economic status, avoidance and approach motivation, threat and challenge appraisal

## Abstract

Social scientists have begun to extensively study how living in contexts with high income inequality affects psychological outcomes. Herein we overview a conceptual framework that integrates, organizes, and extends these complex (and sometimes contradictory) findings. First, we describe studies showing that income inequality breeds an ethos of competitiveness. Second, we argue that the inequality-competitiveness relation explains why income inequality (a) promotes status-focused behaviors aimed at lifting oneself up and/or bringing others down, (b) harms social relations when they pose an obstacle to one’s economic advancement, (c) exerts opposing effects on well-being via avoidance motivation (focusing on the risk of economic failure) and approach motivation (focusing on the prospect of economic success), and (d) represents a threat to those who perceive they do not have sufficient individual/contextual resources to cope with the demands of competition but a challenge to those with sufficient resources. We also discuss limitations and future directions for research.

Over the past 50 years, upper-class income has grown sharply in most countries of the world, while lower-class income has grown modestly, resulting in a historic rise in income inequality (OECD, 2019). Given this global trend, social scientists have begun to study how living in contexts with high income inequality predicts a wide range of psychological outcomes, such as status-focused behaviors, social relations, and well-being (for exemplary work, see Cheung, 2016; Kim et al., 2022; Payne et al., 2017).

However, the scientific literature is arguably one-sided in that many existing literature reviews (a) exclusively portray income inequality as a social ill while ignoring the theoretical reasons why this should not always be the case and (b) disproportionally focus on small cross-sectional studies linking income inequality with negative outcomes while ignoring their methodological limitations (Wilkinson & Pickett, 2017).^1^ Herein, we introduce a conceptual framework—built on the idea that income inequality breeds an ethos of competitiveness—that integrates, organizes, and extends the complex and sometimes contradictory findings of this literature (Fig. 1). We review theoretical and empirical research that illustrates how this framework can be used to not only understand the harmful effects of income inequality but also identify its neutral or even helpful effects. Importantly, we review only high-quality empirical studies with sample sizes sufficient to detect a true small-sized statistical effect of income inequality with a probability of .80 or greater. Moreover, we include only studies that statistically controlled for gross domestic product or an indicator of aggregated income (which is often a confounding factor when examining the link between measures of income inequality and outcomes). Supplemental Table A1 provides a list of these studies and characteristics. This table is publicly available at https://osf.io/5uymx/, along with sensitivity analysis to determine the power of each study.

**Fig. 1.**
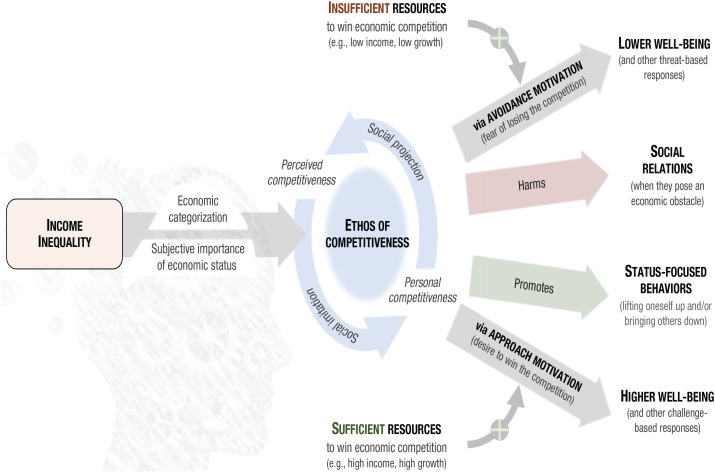
A competitiveness-based theoretical framework on the psychology of income inequality.

## Income Inequality Breeds an Ethos of Competitiveness

By its very definition, income inequality implies greater economic segmentation. In places with high income inequality, the poorest and the richest are further away from one another on the pay scale, making economic differences more readily apparent. This has two immediate, basic cognitive consequences.

First, income inequality increases the relevance of economic categorization. Contrast-weighting theory posits that people pay greater attention to the dimensions in which objects differ rather than the dimensions in which they are similar (Mellers & Biagini, 1994). Applied to society, this means that people from contexts with greater income inequality should give greater weight to the economic dimension (as this dimension is crucial in setting individuals apart from each other). This aligns with the observation that people from these contexts tend to view the world through the prism of wealth, dividing it into the “haves” and the “have-nots” (Peters et al., 2022).

Second, income inequality increases the subjective importance of economic status. Regardless of their culture, humans tend to be highly attuned to their own and others’ position in the social ladder and place particular importance on relative status (Anderson et al., 2015). This tendency is likely rooted in our evolutionary history, although in modern society, status can take on different forms than it used to. As such, when income inequality is high, people specifically ascribe more importance to economic status, and develop concerns for personal success, prestige, and dominance (Du et al., 2022).

As income inequality increases the relevance and subjective importance of economic status, it follows that income inequality breeds an ethos of competitiveness. Both observational and experimental studies have shown that income inequality fuels the perception that those around us are oriented toward competitiveness. For example, in three cross-sectional studies, U.S. residents indicated the degree to which they believed that people compete with one another in their town/city (Sommet et al., 2019). People residing in more economically unequal places systematically perceived their fellow residents as being more competitive. In an experimental replication, participants were introduced to a de novo society with either low or high levels of income inequality (Sánchez-Rodríguez et al., 2019). Participants assigned to the high-inequality condition believed that the cultural climate of that society was more competitive and that individuals behaved more selfishly when sharing resources with a fellow citizen.

**Fig. 2.**
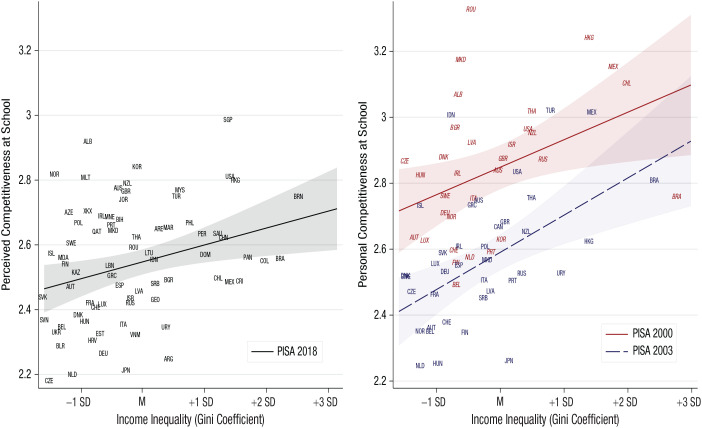
Perceived (left) and personal (right) competitiveness at school as a function of national income inequality (Sommet et al., 2023). Note: The outcome variables use 4-point response scales; the country codes (ISO 3166-1 alpha-3 codes) indicate the national averages of the outcome variable; shaded areas represent 95% confidence intervals.

The fact that income inequality nurtures a culture of positional competition is observed not only among adults but also among school-age children. In three preregistered studies using Programme for International Student Assessment (PISA) data, we recently showed that secondary-school students from economically unequal countries (a) perceived their schoolmates as more competitive and (b) were themselves more competitive (Sommet et al., 2023; see Fig. 2). A fourth preregistered study using an experimental design replicated these findings. The two processes identified in this research are likely reciprocal. As the gap between the poorest and the richest widens and cultural, parental, and/or teachers’ pressure to succeed in stratified societies mounts, students may first develop the perception that others are competitive, which may then prompt personal competitiveness via social contagion (imitating others). Simultaneously, inequality may lead students to first become more competitive, which may then prompt perceived competitiveness via social projection (assuming similarity with others).

## The Psychology of Income Inequality Through the Lens of Competitiveness

The status anxiety hypothesis is arguably one of the most influential frameworks in the psychology of income inequality (Wilkinson & Pickett, 2017). This framework posits that income inequality leads to widespread status anxiety and negative consequences, but it may be incomplete as it does not fully account for the complex and sometimes positive effects of income inequality on psychological outcomes. An alternative approach builds on the robust effect of income inequality on competitiveness to connect the fragmented literature on the psychology of income inequality with various social psychological theories (e.g., social comparison, social interdependence). This approach offers a more comprehensive understanding of the extant findings linking income inequality with behavioral, interpersonal, and well-being outcomes.

### Income inequality prompts status-focused behaviors focused on the self and others

According to social comparison theory (Festinger, 1954), individuals most commonly compare with slightly better-off others, which fuels a desire to remedy the discrepancy between oneself and these others (the pressure toward uniformity). Although many prominent scholars assume that the economic comparisons elicited by income inequality always exert paralyzing effects social comparison theory dictates that such economic comparisons should motivate individuals to narrow the gap (or at least not widen it) between themselves and higher-status others. Specifically, income inequality should give rise to two types of status-focused behaviors: those targeting the self and those targeting others.

First, evidence shows that the ethos of competitiveness engendered by income inequality predicts status-focused behaviors targeting the self (striving to change one’s position to move closer to, or not further away from, higher-status others). As income inequality rises within countries, people from all social classes work longer hours (the “Veblen effect”), presumably to improve/preserve their economic status in comparison to richer reference groups (Alexiou & Kartiyasa, 2020). Moreover, with higher income inequality, people take more economic risks because upward economic comparisons with the rich make them feel they need more to be satisfied (Payne et al., 2017). Finally, residents from more economically unequal localities show greater interest in luxury brands (e.g., Gucci, Rolex), as these goods are used to demonstrate one’s economic status to others (Walasek et al., 2018).

Second, evidence shows that the ethos of competitiveness engendered by income inequality also predicts status-focused behaviors targeting others (striving to change others’ position to reduce, or not increase, the difference in status with them). Two recent field experiments support this proposition. In the first field experiment, pedestrians from deprived neighborhoods exposed to high inequality (through the randomized presence of a high-status car) were more likely to sign a petition to raise taxes on the wealthy (Sands & de Kadt, 2020). This suggests that income inequality leads the poor to want to pull the rich down, so as to minimize economic differences. In addition to striving to bridge the gap between themselves and higher-status others, people may also strive to maintain the gap between themselves and lower-status others (a process that involves downward rather than upward comparison). In the second field experiment, pedestrians from affluent neighborhoods exposed to high inequality (through the randomized presence of a poverty-stricken individual) were less likely to sign a petition to raise taxes on the wealthy (Sands, 2017). This suggests that income inequality leads the rich to want to keep the poor down, so as to maintain economic differences. This dual conclusion is consistent with the rest of the literature: Learning that one’s position on the socioeconomic ladder is lower than one thought *increases* support for redistribution, whereas learning that one’s position is higher than one thought *decreases* support for redistribution, especially when made aware of rising economic inequality (for a review, see Trump, 2021, pp. 89–90).^2^

### Income inequality harms social relations when they pose an economic obstacle

According to social interdependence theory, competitive contexts lead individuals to see others as rivals rather than allies, which is corrosive to social relations (Deutsch, 1949). As income inequality shapes the perception that one’s environment is competitive, it leads people to prioritize their economic self-interest over the welfare of others, which disrupts social bonds. This phenomenon is perhaps best illustrated by a study that compared U.S. census blocks of about one square mile (≈2.5 km^2^) to estimate the association between income inequality and property crime at a fine-grained geographic level (Metz & Burdina, 2018). The findings revealed that income inequality between neighborhoods predicts higher levels of nonviolent profit-driven crime (burglary, larceny, and theft).

Importantly, social comparison exacts a social cost only when the performance dimension is relevant to one’s self-identity (Tesser, 1988). As income inequality fosters the perception of a high-stakes competition over economic resources, it should exact a social cost mainly for economically relevant outcomes. This may explain why people from countries or regions with greater income inequality do not report lower levels of general trust in others (Kim et al., 2022), whereas these individuals do show lower levels of trustworthiness in economic games (i.e., they return less money entrusted to them by another person in an experimental economic game; Johnson & Mislin, 2009; for congruent experimental evidence, see Wei et al., 2022, Study 2).

Also importantly, the type of motivation underlying prosociality matters. Income inequality may paradoxically lead individuals to be more prosocial when it helps them get ahead of the economic competition. For instance, income inequality was found to lead individuals to cooperate with others for instrumental reasons, that is, to form strategic alliances to gain an edge over noncooperating others (Sommet et al., 2023). Income inequality may also lead individuals toward public displays of generosity (e.g., a conspicuous charitable donation) to signal their wealth and earn status (Macchia & Whillans, 2022).

### Income inequality exerts opposing effects on well-being

In the literature, the idea that there is a negative relationship between income inequality and well-being is often presented as a well-established fact (e.g., see Payne et al., 2017). However, there are two reasons to question the nature of this relationship: an empirical reason and a theoretical reason.

From an empirical perspective, most of the influential studies in this area are critically underpowered insofar as they compare only a few dozen countries/regions to establish a relationship between income inequality and well-being (for a study measuring well-being by asking participants whether they are happy, see Oishi et al., 2011; for a study measuring well-being by asking participants whether they are satisfied with their life, see Alesina et al., 2004). In contrast, recent research comparing several hundreds of regions revealed that the relationship between income inequality and well-being was not only statistically indistinguishable from zero but also significantly equivalent to zero (Sommet & Elliot, 2022).^3^

Moreover, from a theoretical perspective, one need not expect the competitiveness elicited by income inequality to be exclusively aversive. Rather, the opposing processes model of competition demonstrates that competitiveness can be experienced as (a) an aversive threat that prompts *avoidance* motivation (being oriented toward the threatening possibility of losing the competition) and (b) an appetitive challenge that prompts *approach* motivation (being oriented toward the promising prospect of winning the competition), with negative and positive implications, respectively (Murayama & Elliot, 2012).

**Fig. 3.**
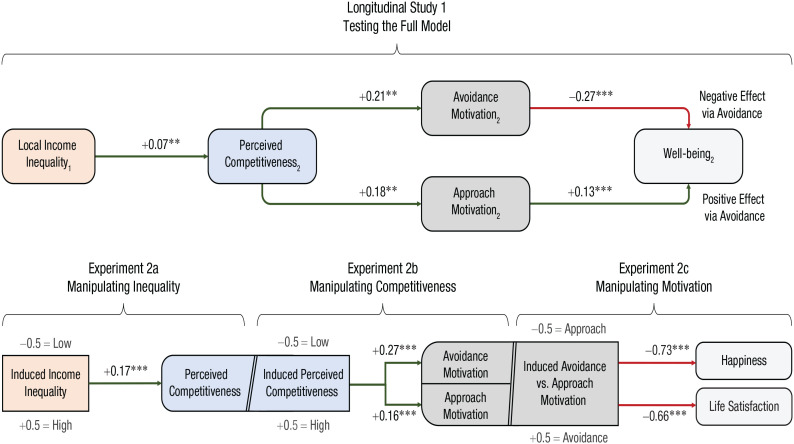
Opposing effects of income inequality on well-being via perceived competitiveness and avoidance/approach motivation (Sommet & Elliot, 2023). Note: Subscripts indicate the wave number. Numbers represent standardized estimates, and values of β = .10, β = .175, and β > .25 can be seen as small, medium, and large effects, respectively. Asterisks indicate significant paths (**p < .01, ***p < .001).

Sommet and Elliot (2023) recently proposed that the absence of an overall link between income inequality and well-being concealed opposing motivational processes. In a 2-year longitudinal study, they found that income inequality predicted perceived competitiveness, which itself (a) negatively predicted well-being via avoidance motivation (sample item: “In general, I am focused on preventing negative events in my life”) and (b) positively predicted well-being via approach motivation (sample item: “I typically focus on the success I hope to achieve in the future”). In three preregistered experiments, they confirmed that income inequality causes an increase in competitiveness, which leads to an increase in both avoidance and approach motivation, which themselves exert negative and positive effects (respectively) on happiness and life satisfaction (Fig. 3).

### Income inequality is a threat for resourceless individuals and a challenge for resourceful individuals

A critical unaddressed question at this point is, “When does income inequality negatively affect well-being, and when does it positively affect well-being?” The biopsychosocial model of challenge and threat tells us that people confronted with a social stressor (an uncertain situation) can react in one of two ways: (a) When they perceive that they do not have enough resources to cope with the stressor, they appraise the situation as a threat, whereas (b) when they perceive that they have enough resources, they appraise it as a challenge (Blascovich & Tomaka, 1996).

Income inequality can be seen as a social stressor because the competitiveness that it breeds creates uneasiness regarding one’s current or future relative economic position. Hence, income inequality should evoke threat and be harmful for people who feel they have insufficient resources to meet the economic competition; conversely, it should evoke challenge and could even be helpful for people who feel they have sufficient resources.

Accordingly, a 15-year longitudinal study documented that the adverse effect of income inequality over time on depression/anxiety is limited to people with insufficient resources to make ends meet (Sommet et al., 2018). Another study reported that income inequality is associated with increased financial hardship for people at the bottom of the income distribution (Jachimowicz et al., 2020), whereas a different study reported that within-country income inequality was associated with decreased status anxiety for people at the top of the distribution (Bartram, 2022).

Resources to cope with economic competition may be located not only at the individual level but also at the structural level. For instance, in contexts with sharp economic growth, income inequality can be interpreted as signaling the potential for upward mobility, generating hope that one will improve their economic status, which increases well-being (Cheung, 2016). Other structural variables, such as equality of opportunity or capability-enhancing policies, could create the perception that economic competition is open and fair, and lessen the threatening nature of income inequality.

## Boundary Condition and Limitations of the Model

An important boundary condition of the theoretical framework presented in this review is that ideological factors may lead individuals to resist the culture of competitiveness promoted by income inequality. In particular, individuals who oppose the idea that social groups should be hierarchically organized (weak social dominance orientation), object to the notion that differences in wealth reflect differences in effort (weak descriptive meritocracy), or disagree that economic success should be achieved through hard work (weak Protestant work ethic) are likely to be impervious to the consequences of competitiveness discussed in our model (for a relevant review, see Willis et al., 2022). Regardless of whether these individuals reject these beliefs for idiosyncratic reasons or because they live in a society where these beliefs are counternormative, they will most likely not engage in status-focused behaviors, damage their relationships, or experience changes in well-being depending on income inequality.

Our model has also two limitations. First, our model does not take into account that inequality can be concentrated at different points in the distribution, such as between the bottom earners and the middle class or between the wealthy and the super-rich (Jachimowicz et al., 2022, pp. 10–12). This means that the effect of income inequality on competition may be confined to the segment of the distribution where inequality is concentrated, specifically leading, for instance, to increased competition among the poor or among the wealthy. Second, our model does not take into account the possibility that the effects of income inequality may be nonlinear. For example, a small increase in income inequality may foster assimilative comparison processes, where resourceful individuals view the status of those who are better off as attainable and become motivated to approach economic success. However, beyond a certain threshold, a large increase in income inequality may foster contrastive comparison processes, where even resourceful individuals view the status of wealthier individuals as unattainable and become discouraged.

## Future Directions and Conclusion

Our model offers three promising avenues for future research:

1. The framework may apply to other outcomes. Income inequality likely predicts threat-based responses among people who feel they have insufficient resources: avoidance-based emotions (e.g., fear), an external economic locus of control, economic self-handicapping, and so on. Conversely, income inequality likely predicts challenge-based responses among people who feel they have sufficient resources: approach-based emotions (e.g., anger), an internal economic locus of control, economic self-entitlement, and so on.
2. The framework may apply to inequality between groups. Just as economic inequality between individuals promotes interpersonal competitiveness, economic inequality between racial, gender, age, or religious groups likely promotes intergroup competitiveness and aversive group outcomes, such as behavioral avoidance, or appetitive intergroup outcomes, such as discrimination (for initial supportive data regarding racial inequality, see Gordils et al., 2020).
3. The framework may apply to noneconomic resources. Inequality of access to various types of desirable resources, such as broader social networks or good grades at school, likely shapes the perception that others compete over these resources and predicts status-focused behaviors to obtain these resources.

In conclusion, we believe that the competitiveness-based framework presented in this review offers a theory-driven, parsimonious, and integrative perspective to better organize the seemingly inconsistent findings of the extant literature on income inequality than perspectives currently available. We hope that this framework will serve as a useful tool for researchers who seek to better understand the psychology of those who live in economically unequal places.

## Recommended Reading

Jachimowicz, J. M., Szaszi, B., Lukas, M., Smerdon, D., Prabhu, J., & Weber, E. U. (2020). (See References). An investigation of the stronger aversive effects of income inequality among the poor.

Peters, K., Jetten, J., Tanjitpiyanond, P., Wang, Z., Mols, F., & Verkuyten, M. (2022). (See References). An article on the basic consequences of income inequality on economic categorization.

Sommet, N., & Elliot, A. J. (2023). (See References). An article of the opposing effects of income inequality on well-being.

Sommet, N., Weissman, D. L., & Elliot, A. J. (2023). (See References). A research article on the effects of income inequality on perceived/personal competitiveness.

Walasek, L., Bhatia, S., & Brown, G. D. (2018). (See References). An analysis of the effects of income inequality on a status-focused behavior.

## Transparency

*Action Editor:* Robert L. Goldstone

*Editor:* Robert L. Goldstone

## Notes

1. “Small cross-sectional studies” are those comparing a small number of units (e.g., countries/regions) at a single point in time. These studies are limited because they have a low power to detect a statistical effect of income inequality and do not enable one to make causal inference.

2. The argument developed in this paragraph is also consistent with recent studies suggesting that income inequality fuels both a fear of being sabotaged by others and a desire for control over others (Cheng et al., 2021; del Fresno-Díaz et al., 2021; both cited in Willis et al., 2022). However, these studies should be considered preliminary because they did not have sufficient statistical power to be formally included in this review.

3. This study used equivalence testing to show that the effect of income inequality was smaller than the smallest negative/positive effects of interest, demonstrating that the effect was equivalent to zero.

## References

[bibr1-09637214231159563] Alesina A. Di Tella R. MacCulloch R. (2004). Inequality and happiness: Are Europeans and Americans different? Journal of Public Economics, 88(9–10), 2009–2042.

[bibr2-09637214231159563] Alexiou C. Kartiyasa A. (2020). Does greater income inequality cause increased work hours? New evidence from high income economies. Bulletin of Economic Research, 72(4), 380–392.

[bibr3-09637214231159563] Anderson C. Hildreth J. A. D. Howland L. (2015). Is the desire for status a fundamental human motive? A review of the empirical literature. Psychological Bulletin, 141(3), 574–601. 25774679 10.1037/a0038781

[bibr4-09637214231159563] Bartram D. (2022). Does inequality exacerbate status anxiety among higher earners? A longitudinal evaluation. International Journal of Comparative Sociology, 63(4), 184–200.

[bibr5-09637214231159563] Blascovich J. Tomaka J. (1996). The biopsychosocial model of arousal regulation. In Zanna M. P. (Ed.), Advances in experimental social psychology (Vol. 28, pp. 1–51). Elsevier.

[bibr6-09637214231159563] Cheung F. (2016). Can income inequality be associated with positive outcomes? Hope mediates the positive inequality-happiness link in rural China. Social Psychological and Personality Science, 7(4), 320–330.

[bibr7-09637214231159563] Deutsch M. (1949). A theory of co-operation and competition. Human Relations, 2(2), 129–152.

[bibr8-09637214231159563] Du H. Götz F. M. King R. B. Rentfrow P. J. (2022). The psychological imprint of inequality: Economic inequality shapes achievement and power values in human life. Journal of Personality. Advance online publication. 10.1111/jopy.12758 35866366

[bibr9-09637214231159563] Festinger L. (1954). A theory of social comparison processes. Human Relations, 7(2), 117–140.

[bibr10-09637214231159563] Gordils J. Sommet N. Elliot A. J. Jamieson J. P. (2020). Racial income inequality, perceptions of competition, and negative interracial outcomes. Social Psychological and Personality Science, 11(1), 74–87.

[bibr11-09637214231159563] Jachimowicz J. M. Davidai S. Goya-Tocchetto D. Szaszi B. Day M. V. Tepper S. J. Phillips L. T. Mirza M. U. Ordabayeva N. Hauser O. P. (2022). Inequality in researchers’ minds: Four guiding questions for studying subjective perceptions of economic inequality. Journal of Economic Surveys. Advance online publication. 10.1111/joes.12507

[bibr12-09637214231159563] Jachimowicz J. M. Szaszi B. Lukas M. Smerdon D. Prabhu J. Weber E. U. (2020). Higher economic inequality intensifies the financial hardship of people living in poverty by fraying the community buffer. Nature Human Behaviour, 4(7), 702–712. 10.1038/s41562-020-0849-2 32231282

[bibr13-09637214231159563] Johnson N. D. Mislin A. (2009). Cultures of kindness: A meta-analysis of trust game experiments. Social Science Research Network working paper series. https://www.mercatus.org/publications/foundational-economic-theory/cultures-kindness-meta-analysis-trust-game-experiments

[bibr14-09637214231159563] Kim Y. Sommet N. Na J. Spini D. (2022). Social class—not income inequality—predicts social and institutional trust. Social Psychological and Personality Science, 13(1), 186–198.

[bibr15-09637214231159563] Macchia L. Whillans A. V. (2022). The link between income, income inequality, and prosocial behavior around the world. Social Psychology, 52(6), 375–386.

[bibr16-09637214231159563] Mellers B. A. Biagini K. (1994). Similarity and choice. Psychological Review, 101(3), 505–518.

[bibr17-09637214231159563] Metz N. Burdina M. (2018). Neighbourhood income inequality and property crime. Urban Studies, 55(1), 133–150.

[bibr18-09637214231159563] Murayama K. Elliot A. J. (2012). The competition-performance relation: A meta-analytic review and test of the opposing processes model of competition and performance. Psychological Bulletin, 138(6), 1035–1070. 23088570 10.1037/a0028324

[bibr19-09637214231159563] OECD. (2019). Under pressure: The squeezed middle class.

[bibr20-09637214231159563] Oishi S. Kesebir S. Diener E. (2011). Income inequality and happiness. Psychological Science, 22(9), 1095–1100. 21841151 10.1177/0956797611417262

[bibr21-09637214231159563] Payne B. K. Brown-Iannuzzi J. L. Hannay J. W. (2017). Economic inequality increases risk taking. Proceedings of the National Academy of Sciences, USA, 114(18), 4643–4648. 10.1073/pnas.1616453114 PMC5422783 28416655

[bibr22-09637214231159563] Peters K. Jetten J. Tanjitpiyanond P. Wang Z. Mols F. Verkuyten M. (2022). The language of inequality: Evidence economic inequality increases wealth category. Personality and Social Psychology Bulletin, 48, 1204–1219. 34350784 10.1177/01461672211036627 PMC9245161

[bibr23-09637214231159563] Sánchez-Rodríguez Á. Willis G. B. Jetten J. Rodríguez-Bailón R . (2019). Economic inequality enhances inferences that the normative climate is individualistic and competitive. European Journal of Social Psychology, 49(6), 1114–1127.

[bibr24-09637214231159563] Sands M. L. (2017). Exposure to inequality affects support for redistribution. Proceedings of the National Academy of Sciences, USA, 114(4), 663–668. 10.1073/pnas.1615010113 PMC5278453 28069960

[bibr25-09637214231159563] Sands M. L. de Kadt D. (2020). Local exposure to inequality raises support of people of low wealth for taxing the wealthy. Nature, 586(7828), 257–261. 32968274 10.1038/s41586-020-2763-1

[bibr26-09637214231159563] Sommet N. Elliot A. J. (2022). The effects of US county and state income inequality on self-reported happiness and health are equivalent to zero. Quality of Life Research, 31(7), 1999–2009. 35482148 10.1007/s11136-022-03137-8 PMC9188529

[bibr27-09637214231159563] Sommet N. Elliot A. J. (2023). Opposing effects of income inequality on health: The role of perceived competitiveness. European Journal of Social Psychology, 53(1), 61–77.

[bibr28-09637214231159563] Sommet N. Elliot A. J. Jamieson J. P. Butera F. (2019). Income inequality, perceived competitiveness, and approach-avoidance motivation. Journal of Personality, 87(4), 767–784. 30284720 10.1111/jopy.12432

[bibr29-09637214231159563] Sommet N. Morselli D. Spini D. (2018). Income inequality affects the psychological health of only the people facing scarcity. Psychological Science, 29(12), 1911–1921. 10.1177/0956797618798620 30312143

[bibr30-09637214231159563] Sommet N. Weissman D. L. Elliot A. J. (2023). Income inequality predicts competitiveness and cooperativeness at school. Journal of Educational Psychology, 115, 173–191.

[bibr31-09637214231159563] Tesser A. (1988). Toward a self-evaluation maintenance model of social behavior. In Berkowitz L. (Ed.), Advances in experimental social psychology (Vol. 21, pp. 181–227). Elsevier.

[bibr32-09637214231159563] Trump K.-S. (2021). Public opinion and reactions to increasing income inequality. In Rosenbluth F. M. Weir M. (Eds.), Who gets what? The new politics of insecurity (pp. 79–102). Cambridge University Press.

[bibr33-09637214231159563] Walasek L. Bhatia S. Brown G. D. (2018). Positional goods and the social rank hypothesis: Income inequality affects online chatter about high-and low-status brands on Twitter. Journal of Consumer Psychology, 28(1), 138–148.

[bibr34-09637214231159563] Wei C. Dang J. Liu L. Li C. Tan X. Gu Z. (2022). Economic inequality breeds corrupt behaviour. British Journal of Social Psychology. Advance online publication. 10.1111/bjso.12610 36444904

[bibr35-09637214231159563] Wilkinson R. G. Pickett K. E. (2017). The enemy between us: The psychological and social costs of inequality. European Journal of Social Psychology, 47(1), 11–24.

[bibr36-09637214231159563] Willis G. B. García-Sánchez E. Sánchez-Rodríguez Á. García-Castro J. D. Rodríguez-Bailón R. (2022). The psychosocial effects of economic inequality depend on its perception. Nature Reviews Psychology, 1, 301–309.

